# Gene family of *Catharanthus roseus* receptor-like kinase 1-like in *Sorghum bicolor*: identification, evolution, function, and stress response

**DOI:** 10.3389/fpls.2026.1751446

**Published:** 2026-03-10

**Authors:** Nana Li, Kai Wang, Hong Xing, Fengfeng Dang, Xiaolong He, Haibin Zhao

**Affiliations:** 1 School of Physical Education, Yan ‘an University, Yan’an, Shaanxi, China; 2 Shaanxi Key Laboratory of Research and Utilization of Resource Plants on the Loess Plateau, College of Life Sciences, Yan’an University, Yan’an, Shaanxi, China; 3 Engineering Research Center of Microbial Resources Development and Green Recycling, University of Shaanxi Province, Yan’an University, Yan’an, Shaanxi, China

**Keywords:** *Catharanthus roseus* receptor-like kinase, drought and salt stresses, duplication events, evolution, SbCrRLK1L gene family

## Abstract

**Introduction:**

The *CrRLK1L* family represents an important subgroup of plant receptor-like kinases (RLKs) that govern growth, signal transduction, reproduction, and stress adaptation.

**Methods:**

In this study, we performed genome-wide identification, phylogenetic reconstruction, gene structure and motif analysis, evolutionary duplication analysis, promoter cis-element prediction, and tissue- and stress-specific expression profiling of *SbCrRLK1L* genes.

**Results:**

A total of 28 *SbCrRLK1L* genes were identified and clustered into four well-supported subgroups with conserved structural features. Multiple duplication events, including WGD, TD, PD, DSD, and TRD, contributed to the expansion of this gene family. Promoter analysis revealed abundant cis-elements associated with hormonal regulation, stress responses, and development. Expression analysis showed that *SbCrRLK1L1/8/17*/24/25/26 were predominantly expressed in roots, while *SbCrRLK1L1/8/17*/24/25 were significantly regulated by drought and salt stress.

**Discussion:**

The expression of specific *SbCrRLK1L* genes suggests their potential roles in root development. The strong transcriptional responsiveness to abiotic stress indicates that key *SbCrRLK1L* members may act as critical regulators in sorghum stress tolerance. Collectively, our findings provide a foundation for dissecting the functions of *CrRLK1L* genes in sorghum development and stress adaptation.

## Introduction

The Catharanthus roseus receptor-like kinase 1-like (*CrRLK1L*) gene family belongs to the receptor-like kinase (RLK) superfamily and plays crucial roles in regulating plant growth, developmental processes, signal transduction, and stress adaptation (Shiu and Bleecker, 2001; Zhu et al., 2021; Franck and Westermann, 2018; Feng et al., 2018). Proteins encoded by CrRLK1L genes typically share a conserved structure, comprising an extracellular domain capable of perceiving external signals, a single-pass transmembrane domain, and an intracellular kinase domain that transmits signals through phosphorylation (Liu et al., 2024). The extracellular domain senses external signals, the transmembrane domain facilitates the transmission of signals into the cell, and the intracellular kinase domain activates downstream signaling pathways through phosphorylation (Kanaoka and Torii, 2010; Stegmann et al., 2017; Solis and Quinto, 2021).

In *Arabidopsis thaliana*, 17 *CrRLK1L* members have been identified (Hématy et al., 2009). Among them, FERONIA (FER) serves as a central multifunctional signaling hub, integrating inputs from various ligands and hormones to regulate growth, development, and stress responses (Duan et al., 2010; Li et al., 2015; Schoenaers et al., 2018; Dong et al., 2019; Zhu et al., 2020; Zhong et al., 2022; Duan et al., 2020). During growth and development, FER plays a central role in regulating essential processes, including cell elongation, pollen tube guidance and rupture, fertilization, and root hair development, by binding to its extracellular ligand, the Rapid Alkalization Factor (RALF) family of peptides (Duan et al., 2010; Li et al., 2015; Schoenaers et al., 2018; Dong et al., 2019; Zhu et al., 2020; Zhong et al., 2022; Duan et al., 2020). For example, during fertilization, FER precisely controls pollen tube reception by regulating the accumulation of low-methylated pectin and nitric oxide at the micropyle (Duan et al., 2010). Studies indicate that FER interacts with ABI2-type phosphatases, establishing crosstalk between abscisic acid (ABA) signaling and RALF peptide signaling (Chen et al., 2016). The protein module comprising leucine-rich repeat-expanded (LRX) proteins, RALF peptides, and FER receptors has been shown to coordinate multiple plant hormone signaling pathways and regulate plant growth and adaptation to salt stress (Zhao et al., 2020). Furthermore, FER modulates the stability of serine hydroxymethyltransferase 1 (SHM1) through phosphorylation. This influences metabolic flux in the photorespiration pathway, ultimately aiding plant adaptation to high-salinity environments (Jiang et al., 2024). In addition to FER, other members of the *A. thaliana* CrRLK1L family have important functions. For instance, HERK1 and ANX1/ANX2 have been reported to interact synergistically with FER and play a role in maintaining the integrity of the pollen tube cell wall (Liu et al., 2021). In immune responses, the receptor kinase LLG1 interacts with FLS2 and EFR, activating RbohD downstream to promote reactive oxygen species (ROS) production (Shen et al., 2017). Recent studies have also revealed a signaling axis named PHR1-RALF-FERONIA. This pathway recruits specific members of the root microbiome to help plants alleviate phosphorus starvation stress by upregulating the expression of phosphorus starvation response (PSR) genes (Tang et al., 2022).

In *Oryza sativa*, 16 *CrRLK1L* genes have been reported. Research indicates that the OsRALF26-OsFLR1 module functions within the XA21-mediated immune pathway, activating defense responses against bacterial pathogens (Kwon et al., 2024). Furthermore, OsFLR1 and OsFLR29 are implicated in silicon uptake and accumulation, suggesting a role in nutrient homeostasis (Lei et al., 2025). Under abiotic stress, the malectin-like receptor kinase OsMRLK63 positively regulates drought tolerance by modulating ROS production via the OsRALF45/46–OsMRLK63–OsRbohs signaling pathway (Jing et al., 2024).

In *Zea may*), 23 *CrRLK1L* members were identified (Wang et al., 2024), with ZmFLR1, ZmFLR2, and ZmFLR3 contributing to broad-spectrum disease resistance against pathogens causing northern leaf blight, anthracnose stem rot, and southern leaf blight (Yu et al., 2022). In *Solanum lycopersicum*, which possesses 24 CrRLK1-like members (Ma et al., 2023a), a RALF2–FERONIA–MYB63 module fine-tunes the balance between root growth and defense by regulating lignin deposition, influencing resistance to *Fusarium oxysporum* (Fan et al., 2024). Additionally, the tomato BZR1 transcription factor interacts with FERONIA-mediated ROS signaling to enhance heat tolerance, revealing a novel mechanism for CrRLK1Ls in abiotic stress adaptation (Yin et al., 2018).

Although the *CrRLK1L* gene family has been systematically characterized in model plants like *A. thaliana* and *O. sativa*, as well as in crops like *Z. mays* and *S. lycopersicum* a comprehensive genome-wide analysis of this family in *S. bicolor* is still lacking. *S. bicolor* is a vital cereal crop renowned for its exceptional drought and salinity tolerance (Chen et al., 2025). This study aims to fill this knowledge gap by conducting a genome-wide identification and characterization of the *CrRLK1L* gene family in *S. bicolor*. We analyze their phylogenetic relationships, gene structures, conserved motifs, chromosomal locations, duplication events, and promoter cis-elements. Furthermore, we investigate their expression profiles across different tissues and under drought and salt stress conditions. Our findings provide a foundation for elucidating the biological functions of *S. bicolor CrRLK1L* genes and their potential applications in enhancing stress resistance in crops.

## Methods and materials

### *SbCrRLK1L* gene identification

The *S. bicolor* genome sequence and annotation files were downloaded from the Phytozome v13 database (https://phytozome-next.jgi.doe.gov/) (Goodstein et al., 2012). To identify putative *SbCrRLK1L* genes, firstly, the HMM (Hidden Markov Model) of Malectin_like (PF12819) and Pkinase_Tyr (PF07714) were obtained from the Pfam database (Mistry et al., 2021) (https://pfam.xfam.org/) , and use HMMER software (E-value<1e-5) to search for *SbCrRLK1L* genes in the *S. bicolor* protein database. Secondly, according to the BLASTP method (E-value<1e-5), we searched *SbCrRLK1L* protein sequences using At*CrRLK1L* protein sequences. Firstly, we retained the members with an amino acid sequence length greater than 400 amino acids. This threshold was set based on the lengths of multiple reported CrRLK1L proteins (for example, the length range of *Solanum lycopersicum* CrRLK1L members is 811-1340 aa (Ma et al., 2023a); the length range of *Solanum melongena* members CrRLK1L is 443-1084 aa (Ma et al., 2023b)). This threshold was established to initially exclude “pseudo-genes” caused by genomic annotation errors, poor sequencing quality, or incomplete gene models. Candidate genes obtained from the initial search were subsequently subjected to domain analysis using the Pfam Database (Mistry et al., 2021) and SMART databases to verify the presence of the conserved CrRLK1L domain. Only those proteins that contained the characteristic CrRLK1L domain were ultimately designated as members of the *S. bicolor CrRLK1L* gene family.

### Chromosomal localization and physicochemical property analysis

The physicochemical properties of the protein sequences encoded by the identified *S. bicolor CrRLK1L* gene family were analyzed using TBtools software (Chen et al., 2023). This in *silico* analysis predicted key parameters, including molecular weight, theoretical isoelectric point (pI), amino acid composition, instability index, and grand average of hydropathicity (GRAVY).

Based on the physical location information extracted from the *S. bicolor* genome annotation file, the chromosomal positions of the CrRLK1L gene family members were mapped using TBtools software (Chen et al., 2023). The resulting distribution map visually presents the locations of these genes across the *S. bicolor* chromosomes, facilitating analysis of their distribution characteristics and potential clustering patterns.

### Phylogenetic analysis

To investigate the evolutionary relationships of the *CrRLK1L* gene family, a phylogenetic analysis was conducted using amino acid sequences from *S. bicolor* (obtained in this study), *O. sativa*, *Z. mays*, and *A. thaliana*. The full-length amino acid sequences of CrRLK1L members from these species were retrieved from the Phytozome v13 database (Goodstein et al., 2012). Multiple sequence alignment was performed using ClustalW software with default parameters. A phylogenetic tree was subsequently constructed based on the aligned sequences using the Neighbor-Joining (NJ) method implemented in MEGA 7 software (Kumar et al., 2016). The robustness of the inferred tree topology was assessed by bootstrap analysis with 1,000 replicates.

### Gene structure and conserved motif analysis

The exon-intron structures of the identified *CrRLK1L* gene family members were analyzed based on the *S. bicolor* genome annotation file using TBtools software (Chen et al., 2023). Conserved protein motifs were identified using the online tool MEME (Bailey et al., 2009), with the motif width set to 6–50 amino acids and other parameters retained as defaults. The visualization of the predicted motifs, along with the exon-intron structures, was integrated and graphically presented using TBtools (Chen et al., 2023) to facilitate an investigation of potential correlations between motif composition and gene function.

### Duplication events and Ka/Ks analysis

To investigate the duplication events and evolutionary constraints acting on the *S. bicolor CrRLK1L* gene family, intra-genomic synteny analysis was performed using MCScanX software (Wang et al., 2012). This analysis identified collinear blocks within the *S. bicolor* genome to assess segmental and tandem duplication events among the family members. For gene pairs involved in these duplication events, the non-synonymous (Ka) and synonymous (Ks) substitution rates were calculated using the KaKs_Calculator function integrated within TBtools software (Chen et al., 2023). The resulting Ka/Ks ratios were interpreted to infer the mode of selection pressure: Ka/Ks < 1, = 1, and > 1 indicating purifying selection, neutral evolution, and positive selection, respectively.

### Analysis of promoter cis-acting elements

Promoter sequences were extracted from the *S. bicolor* genome by selecting the 2,000-bp region upstream of the start codon for each *CrRLK1L* gene family member. The sequences were then submitted to the PlantCARE online database (https://bioinformatics.psb.ugent.be/webtools/plantcare/html/Menu.html) for analysis of cis-acting elements (Lescot et al., 2002).

### tissue expression pattern analysis

To systematically analyze the tissue-specific expression patterns of the *S. bicolor CrRLK1L* gene family, multi-tissue transcriptome data encompassing various developmental stages were obtained from the *Sorghum* Genomics and Mutation Database (SGMD) (https://sorghum.genetics.ac.cn/SGMD/Expression.html) (Chen et al., 2025). This database offers a telomere-to-telomere (T2T), gap-free reference genome for the inbred line E048, along with high-quality gene expression profiles. The dataset included key tissues such as root, stem, leaf, and inflorescence. Gene expression levels, normalized as Transcripts Per Million (TPM), were extracted for each *CrRLK1L* member using TBtools software. Finally, a heat map was generated to visualize the distinct expression patterns across tissues.

### Plant materials and abiotic stress treatments

The standard inbred *S. bicolor* line BTx623 was selected for this study. Seeds were surface-disinfected by immersion in a 1% (v/v) sodium hypochlorite solution for 10 minutes, followed by three rinses with sterile distilled water. Subsequently, the seeds were placed on moist filter paper in Petri dishes and germinated in a growth chamber maintained at 28 °C/25 °C (day/night), under a 16-h light/8-h dark photoperiod and 70% relative humidity. Uniformly germinated seedlings at the two-leaf stage were selected and transferred to hydroponic systems containing half-strength Hoagland nutrient solution, which was renewed daily.

When the seedlings reached the three-leaf stage, uniformly developed individuals were subjected to stress treatments. The stress concentrations employed in different studies vary considerably (e.g., 150–300 mmol/L NaCl, 15–30% PEG-6000) (Yao et al., 2023; Shi et al., 2025). Based on the results of previous experiments, we found that the stress treatment of 200 mmol/L NaCl and 20% PEG-6000 could effectively induce significant responses to osmotic stress and drought stress in *S. bicolor*, and at the same time provided a stable and repeatable experimental window for observing changes in gene expression. Therefore, in this study, we finally selected 200 mmol/L NaCl and 20% PEG-6000 as the treatment concentrations to simulate salt stress and drought stress. The experiment included three groups: (1) a control group (CK) maintained in half-strength Hoagland nutrient solution; (2) a salt-stress group (NaCl) exposed to half-strength Hoagland solution supplemented with 200 mmol/L NaCl; and (3) a drought-stress group (PEG6000) treated with half-strength Hoagland solution containing 20% (w/v) PEG6000 to simulate moderate drought stress.

Root samples were collected at 0 and 48 hours after stress application. The samples were rapidly frozen in liquid nitrogen and stored at -80 °C for subsequent RNA extraction and gene expression analysis.

### RNA extraction and RT-qPCR

Total RNA was isolated from *S. bicolor* root tissues using the KKFast Plant RNApure Kit. The extracted RNA served as the template for genomic DNA removal and first-strand cDNA synthesis, which was performed with the TaKaRa PrimeScript™ RT Reagent Kit with gDNA Eraser. The procedure involved two main steps: first, residual genomic DNA was eliminated by incubation at 42 °C for 5 min in the gDNA Eraser reaction system; subsequently, reverse transcription was carried out using the PrimeScript RT enzyme mix at 37 °C for 30 min, followed by enzyme inactivation at 85 °C for 5 s. The synthesized cDNA was diluted fivefold with sterile water and stored at −20 °C for subsequent experiments.

Quantitative real-time PCR (RT-qPCR) was conducted using SYBR Premix Ex Taq™ on a real-time PCR system. Each 10 μL reaction mixture contained 5 μL of SYBR Premix Ex Taq, 0.5 μL of forward primer (10 μM), 0.5 μL of reverse primer (10 μM), 1 μL of cDNA template, and 3 μL of ddH_2_;O. The amplification program consisted of an initial denaturation at 95 °C for 30 s, followed by 40 cycles of denaturation at 95 °C for 5 s and annealing/extension at 60 °C for 30 s.

Gene expression levels were calculated using the 2^-ΔΔCt^ method. We used the *ACTIN* gene, which was stably expressed at each growth stage in almost all tissues, as an internal control (Yao et al., 2023; Liu et al., 2025). Each experiment included three independent biological replicates. Statistical analysis and visualization of RT-qPCR data were performed using GraphPad Prism 9.0 software. Significant differences between treatments were determined by one-way analysis of variance (ANOVA), and error bars represent the standard error of the mean from three replicates.

## Results

### Identification of CrRLK1L gene family members in *S. bicolor*

**Figure 1:**
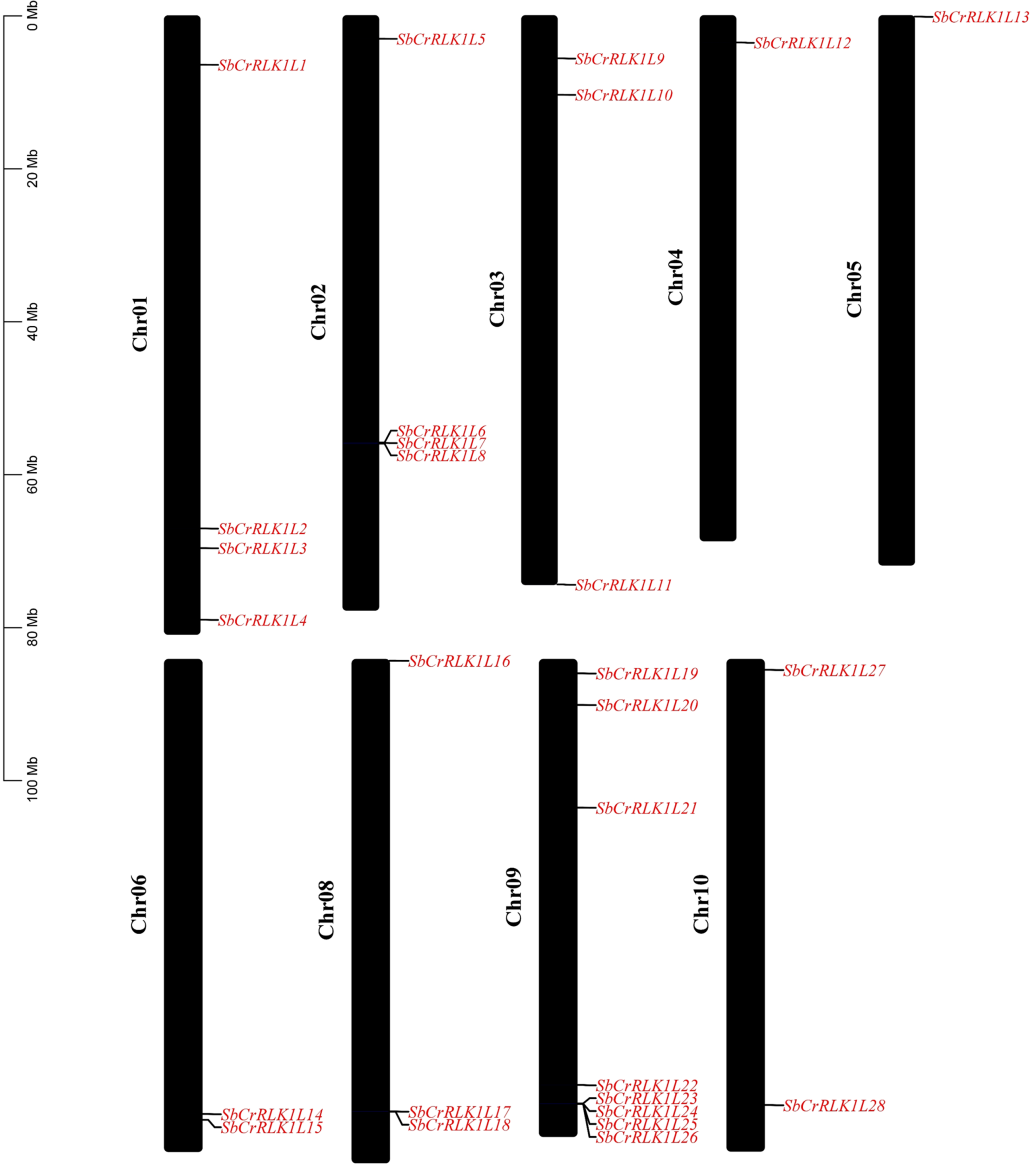
Chromosomal mapping of members of the *S. bicolor CrRLK1L* gene family.

A total of 28 *CrRLK1L* gene family members were identified in the *S. bicolor* genome through a combination of Hidden Markov model (HMM) profiling and bidirectional BLAST analysis. Following established nomenclature, these genes were designated *SbCrRLK1L1* to *SbCrRLK1L28* according to their physical positions on the chromosomes (**Figure 1** and **Supplementary Table 1**). Chromosomal location analysis revealed an uneven distribution of these genes across the nine *S. bicolor* chromosomes. A notable clustering pattern was observed for specific gene subsets; for instance, three genes (*SbCrRLK1L6*, *SbCrRLK1L7*, and *SbCrRLK1L8*) are tightly arranged on chromosome 2, and another cluster of four genes (*SbCrRLK1L23*, *SbCrRLK1L24*, *SbCrRLK1L25*, and *SbCrRLK1L26*) was identified on chromosome 9 (**Figure 1**). This clustered genomic arrangement suggests that segmental or tandem duplication events may have contributed to the expansion of this gene family, and these physically linked members may potentially function cooperatively in related biological processes.

**Figure 2:**
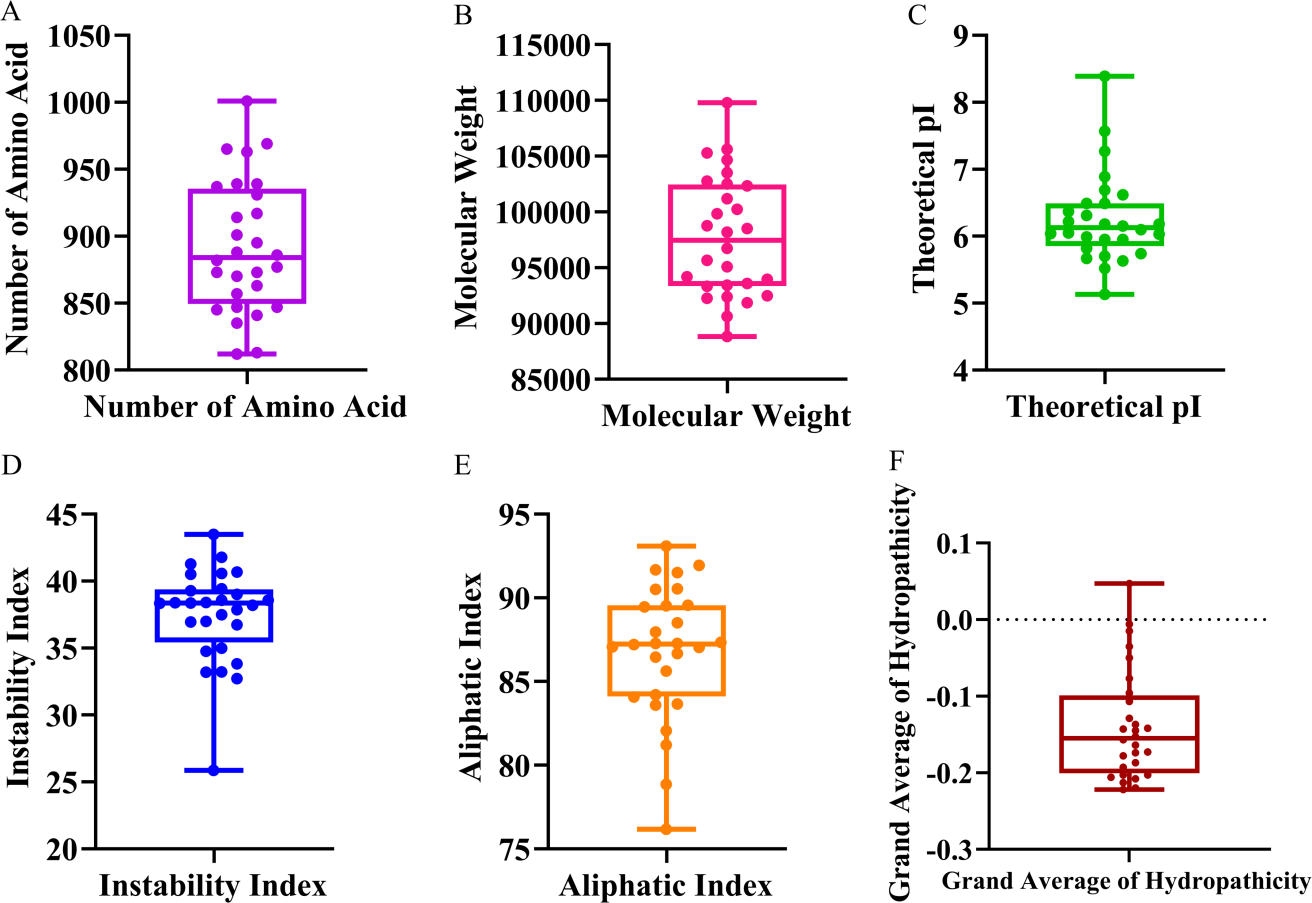
Physicochemical Properties of Proteins the SbCrRLK1L Gene Family. **(A)** Amino acid sequence length distribution of **(S)** bicolor CrRLK1L proteins. **(B)** Molecular weight range of the encoded proteins. **(C)** Theoretical isoelectric point (pI) values. **(D)** Instability index. **(E)** Aliphatic Index. **(F)** Grand average of hydropathicity (GRAVY) values.

The physicochemical properties of the 28 identified *S. bicolor* CrRLK1L proteins were systematically characterized. The amino acids length ranged from 812 (SbCrRLK1L4) to 1,001 (SbCrRLK1L3) (**Figure 2A**), corresponding to molecular weights between 88.83 kDa and 109.78 kDa (**Figure 2B**). Theoretical isoelectric point (pI) values exhibited a broad spectrum from 5.52 (SbCrRLK1L18) to 8.39 (SbC2ArRLK1L22), with the majority (89.29%) of proteins possessing acidic pIs (<7), while only three members (SbCrRLK1L19, SbCrRLK1L22, and SbCrRLK1L28) had basic pIs (>7) (**Figure 2C**). Instability index indicated that 78.57% of the proteins displayed instability indices below 40, suggesting a propensity for stable structural conformations (**Figure 2D**). Aliphatic Index varied from 76.18 to 93.1, reflecting general hydrophobic characteristics (**Figure 2E**). However, evaluation of the grand average of hydropathicity (GRAVY) showed that most proteins were hydrophilic, with SbCrRLK1L5 being the sole exception exhibiting hydrophobic properties (**Figure 2F**). These pronounced differences in intrinsic physicochemical parameters among *S. bicolor* CrRLK1L members imply significant functional diversification within this gene family.

### Phylogenetic analysis

**Figure 3:**
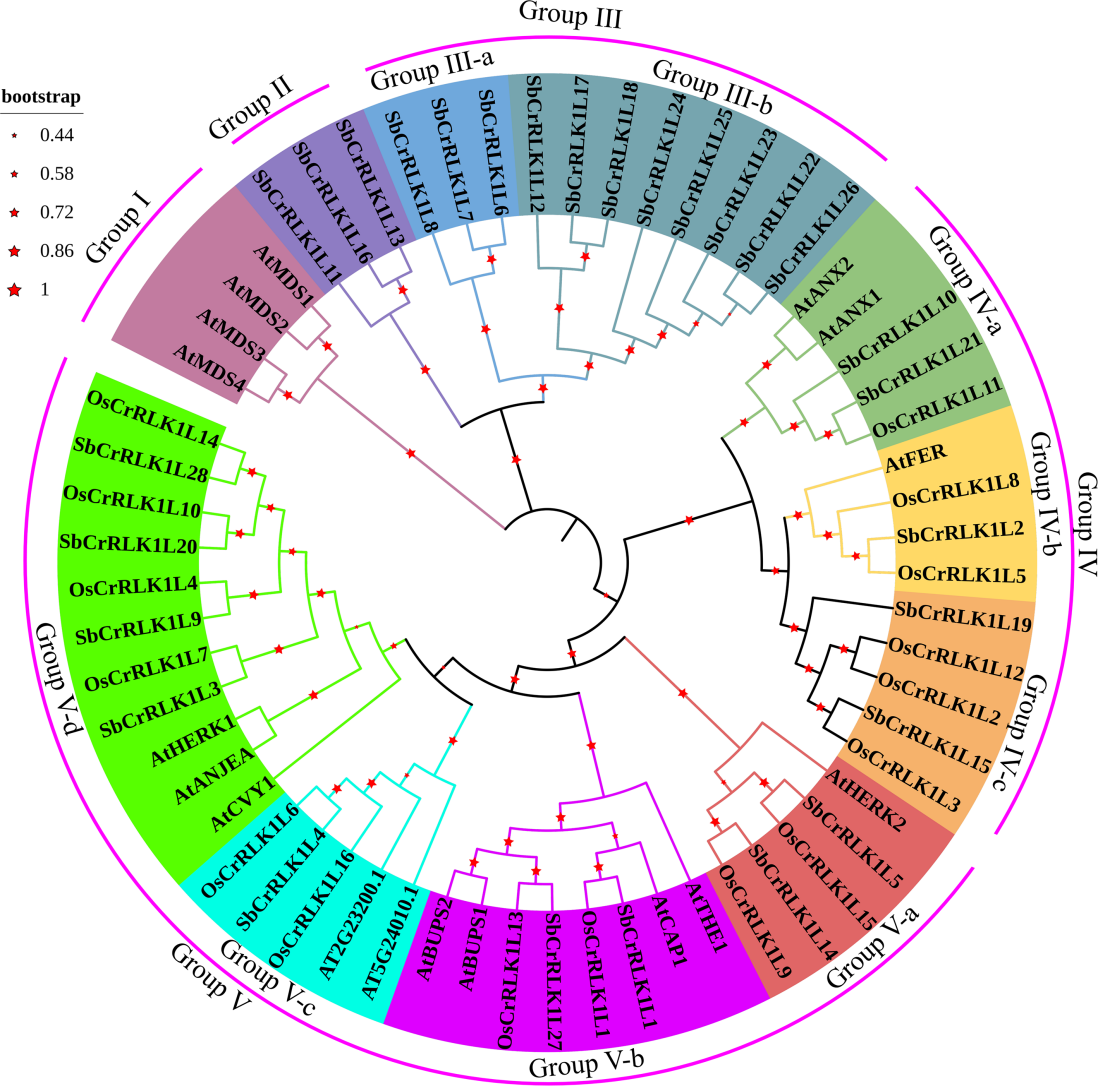
Phylogenetic tree of the *CrRLK1L* gene family in *S. bicolor*, *O. sativa*, and *A. thaliana*. This evolutionary tree was generated through MEGA 7 software (method: neighbor-joining; bootstrap: 1000). Different colors represent different subgroups.

To elucidate the evolutionary relationships among *CrRLK1L* gene family members, a phylogenetic tree was constructed using the neighbor-joining method in MEGA 7 software, based on the full-length protein sequences of *CrRLK1L* genes from *S. bicolor*, *O. sativa*, and *A. thaliana*. Presented in **Figure 3**, these members are classified into five distinct monophyletic subgroups (Group I to Group V). Analysis of the tree topology revealed that *CrRLK1L* genes from *S. bicolor* and *O. sativa* consistently clustered together within the same major branches, reflecting their close phylogenetic relationship as members of the Poaceae family. In contrast, *S. bicolor* and *A. thaliana* members were distributed across divergent branches (**Figure 3**), indicating a more distant evolutionary relationship. Further examination showed that Group II and Group III are specific to *S. bicolor*, whereas Group I comprises exclusively *A. thaliana* members. Notably, Group III could be further subdivided into two well-supported subclades (Group III-a and III-b), suggesting potential functional divergence or distinct evolutionary origins within this subgroup. A significant finding was that the *S. bicolor* gene SbCrRLK1L2 and the well-characterized FERONIA (AtFER) reside within the same subfamily and exhibit a close phylogenetic relationship, implying they may share conserved biological functions related to growth, stress response, and signal transduction.

### Gene structure and conserved motifs

**Figure 4:**
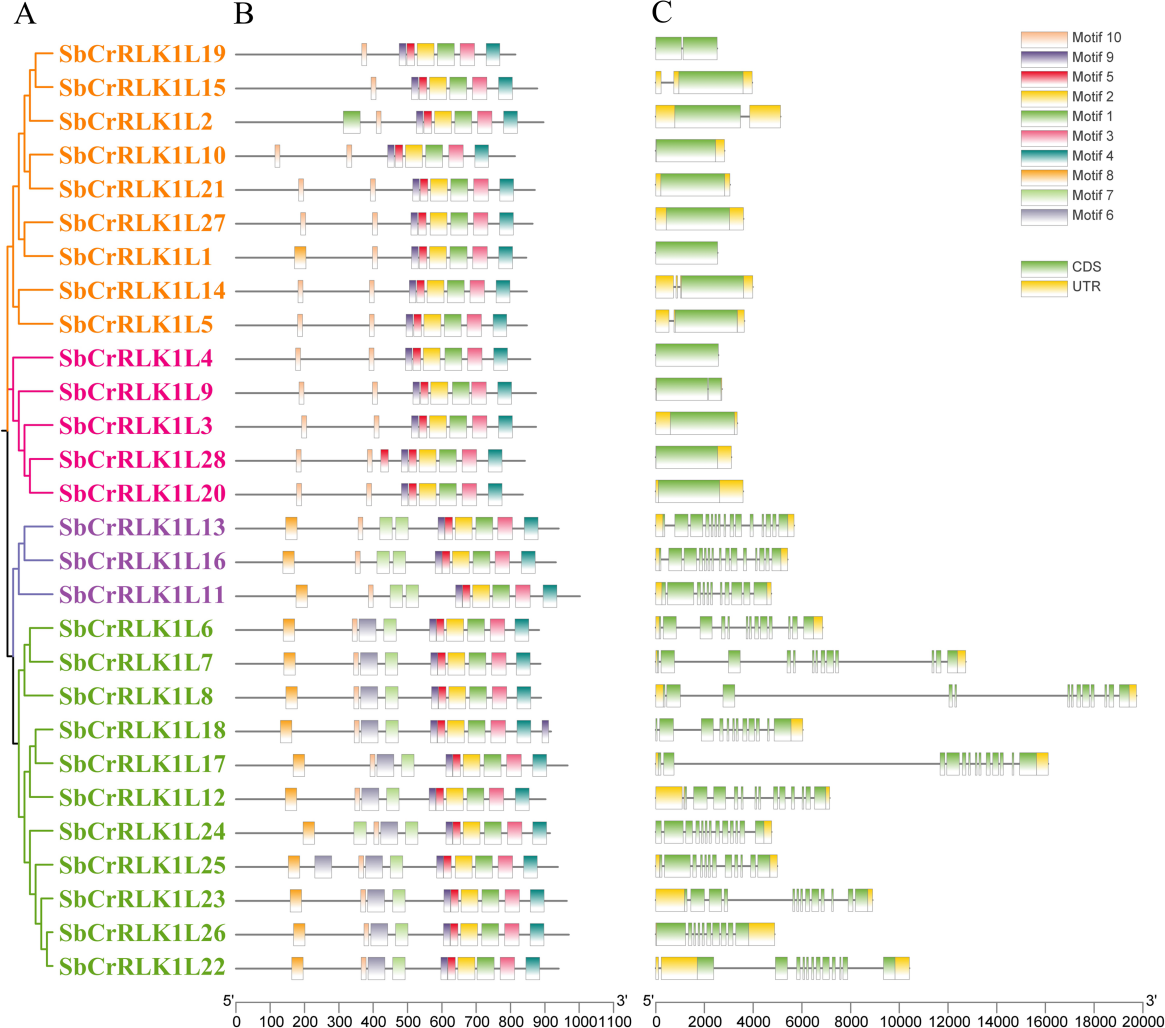
Gene Structure and Conserved Motif Composition of *S. bicolor CrRLK1L* Family Members. **(A)** Distribution of ten conserved protein motifs identified by MEME analysis. Distinctly colored rectangles represent unique motifs. **(B)** Exon-intron structure schematic. Green rectangles indicate exons, gray lines connecting them represent introns.

Conserved motif and gene structure analyses provide important insights into the functional diversification of the *S. bicolor CrRLK1L* gene family. Using the MEME online tool, ten conserved motifs were identified among the *S. bicolor* CrRLK1L proteins (**Figures 4A, B**). Although the type and number of motifs varied across different members, several motifs exhibited high conservation. Specifically, motifs 1, 2, 3, 4, 9, and 10 were consistently present in the vast majority of *CrRLK1L*, suggesting their potential role in core functional mechanisms. Furthermore, the distribution of certain motifs was subgroup-specific (**Figures 4A, B**). For instance, motifs 6, 7, and 8 were predominantly found in members belonging to Group I and Group II, indicating that these motifs may confer specialized functions to these particular subgroups.

Analysis of the exon-intron structure using TBtools software revealed substantial architectural diversity among the *S. bicolor CrRLK1L* genes (**Figure 4C**). Specifically, Group I and Group II genes possessed complex structures, containing between nine and fourteen introns. In contrast, Group III and Group IV displayed comparatively simpler architectures, harboring only one to three introns. These findings provide valuable insights into the potential functional diversification of *S. bicolor CrRLK1L* family genes.

### Duplication events and Ka/Ks analysis

**Figure 5:**
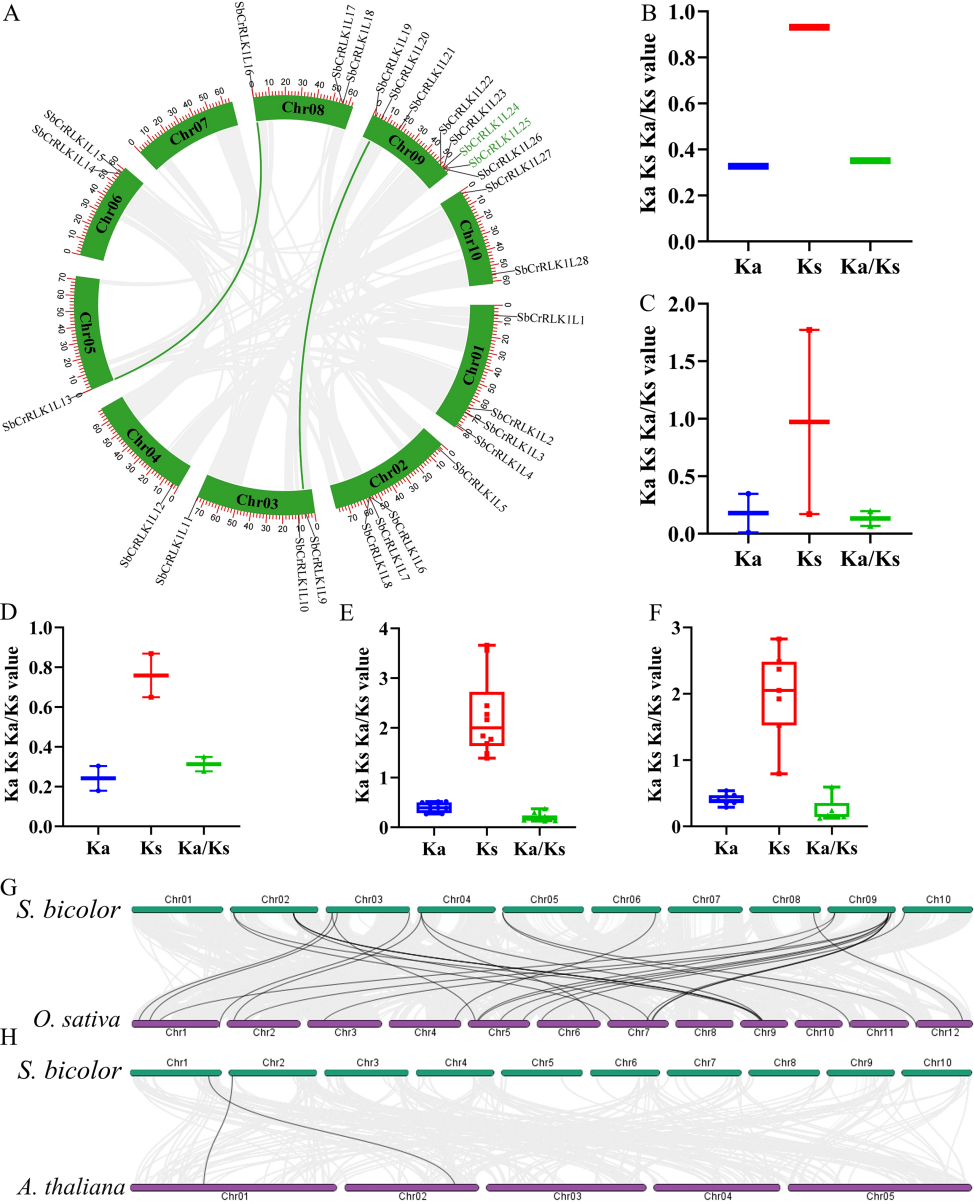
Gene Duplication Events and Evolutionary Selection Analysis of the *S. bicolor CrRLK1L* Gene Family. **(A)** Types and distribution of gene duplication events. Green lines connect gene pairs resulting from whole-genome duplication (WGD), while green gene labels highlight tandem duplication pairs. **(B–F)** Evolutionary constraint analysis of duplicated gene pairs. Ka (non-synonymous substitution rate), Ks (synonymous substitution rate), and Ka/Ks ratios were calculated for each duplication type: TD **(B)**, WGD **(C)**, PD **(D)**, TRD **(E)**, and DSD **(F)** duplicates. **(G–H)** Cross-species synteny analysis. **(G)** Collinearity between *S. bicolor* and *O. sativa CrRLK1L* genes. Gray lines connect 28 syntenic gene pairs. **(H)** Collinearity between *S. bicolor* and *A. thaliana CrRLK1L* genes.

A comprehensive analysis of the *S. bicolor* genome using MCScanX software revealed that the expansion of the *CrRLK1L* gene family was driven by multiple modes of gene duplication. These types include whole-genome duplication (WGD), tandem duplication (TD), proximal duplication (PD), dispersed duplication (DSD) and transposed duplication (TRD). Specifically, two WGD gene pairs, one TD gene pair, three PD gene pairs, ten TRD gene pairs, and nine DSD gene pairs were identified (**Figure 5A** and **Supplementary Table 2**). For instance, *SbCrRLK1L24/SbCrRLK1L25* were characterized as a tandem duplicate pair, while *SbCrRLK1L13/SbCrRLK1L16* and *SbCrRLK1L9/SbCrRLK1L20* were identified as WGD gene pairs. An interesting “one-to-many” duplication pattern was observed for the *SbCrRLK1L2*, which showed syntenic relationships with multiple paralogs (*SbCrRLK1L14*, *SbCrRLK1L15*, *SbCrRLK1L21*, and *SbCrRLK1L27*), suggesting it may have served as a progenitor for several duplication events during evolution.

To assess the selective pressures acting on the duplicated gene pairs, the non-synonymous (Ka) and synonymous (Ks) substitution rates were calculated using Tbtools. The resulting Ka/Ks ratios for all duplicated gene pairs were less than 1 (**Figures 5B–F** and **Supplementary Table 2**), indicating that the *S. bicolor CrRLK1L* gene family has predominantly undergone purifying selection throughout its evolution. This selective constraint favors the preservation of ancestral gene functions and maintains functional stability among paralogs.

A comparative genomic analysis was conducted to evaluate the evolutionary conservation of the CrRLK1L family across species. Genome-wide synteny assessment revealed 28 collinear gene pairs between *S. bicolor* and *O. sativa*, but only two pairs between *S. bicolor* and *A. thaliana* (**Figures 5G, H**). This disparity underscores a higher degree of conservation between *S. bicolor* and *O. sativa*, consistent with their closer evolutionary relationship within the Poaceae family. Furthermore, the identification of “one-to-many” collinear relationships, such as the association of *S. bicolor SbCrRLK1L9* with both *OsCrRLK1L4* and *OsCrRLK1L10* in *O. sativa*, implies that lineage-specific duplication events occurred in the *O. sativa* genome after it diverged from a common ancestor with *S. bicolor*. These newly acquired gene copies may have subsequently undergone functional diversification, contributing to the functional adaptation of the CrRLK1L family within the grass lineage.

### Analysis of promoter cis-acting elements

**Figure 6:**
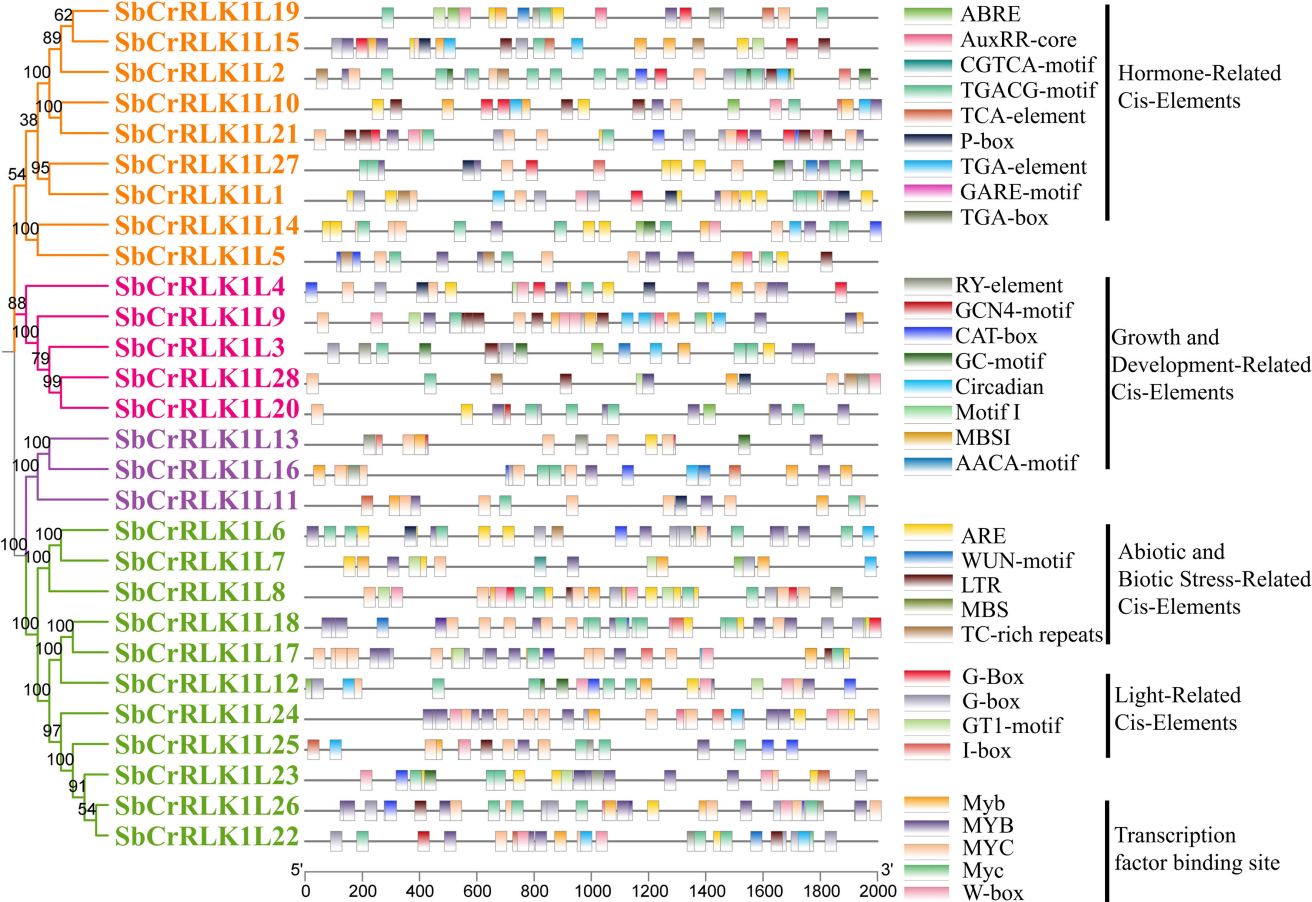
Analysis of cis-acting elements in the promoters of *S. bicolor CrRLK1L* gene family members.

To elucidate the transcriptional regulatory mechanisms of the *S. bicolor CrRLK1L* gene family, this study extracted a 2,000 bp genomic sequence upstream of the start codon for each member as the promoter region. The *cis*-acting elements were performed using the PlantCARE database. The results revealed that all promoters contained an abundance of regulatory elements, which were systematically classified into three primary categories: 1) hormone-responsive elements, including the abscisic acid response element (ABRE) and the auxin response element (AuxRR-core); 2) stress-responsive elements, such as the drought-inducible element (MBS) and the low-temperature response element (LTR); and 3) elements associated with developmental processes, such as circadian rhythm regulation (circadian) and seed-specific expression (RY-element) (**Figure 6** and **Supplementary Table 3**).

Notably, the promoters of several genes were found to harbor combinations of stress- and hormone-related elements. For instance, the promoters of *SbCrRLK1L1*, *SbCrRLK1L3*, *SbCrRLK1L7*, *SbCrRLK1L8*, and *SbCrRLK1L5*concurrently contained both ABRE and MBS elements, suggesting their potential involvement in ABA-mediated drought stress response pathways. Furthermore, the promoters of genes such as *SbCrRLK1L1*, *SbCrRLK1L4*, *SbCrRLK1L6*, and *SbCrRLK1L11* were enriched with multiple hormone-responsive motifs, including ABRE, CGTCA-motif (linked to jasmonic acid response), and P-box (associated with gibberellin response), indicating a potential for complex co-regulation by abscisic acid, jasmonic acid, and gibberellin signaling pathways.

Additionally, the presence of the drought-responsive MBS element was widespread, being identified in the promoters of *SbCrRLK1L1*, *SbCrRLK1L2*, *SbCrRLK1L8*, *SbCrRLK1L14*, *SbCrRLK1L17*, *SbCrRLK1L24*, *SbCrRLK1L25*, and *SbCrRLK1L126* (**Figure 6**), underscoring a likely crucial role for the *CrRLK1L* gene family in *S. bicolor* adaptive response to water deficit. The prevalence of these diverse *cis*-acting elements suggests that the *S. bicolor CrRLK1L* gene family is subject to complex transcriptional regulation, potentially integrating hormonal signals and stress cues to modulate key physiological processes during growth, development, and environmental adaptation.

### Analysis of tissue-specific expression patterns

**Figure 7:**
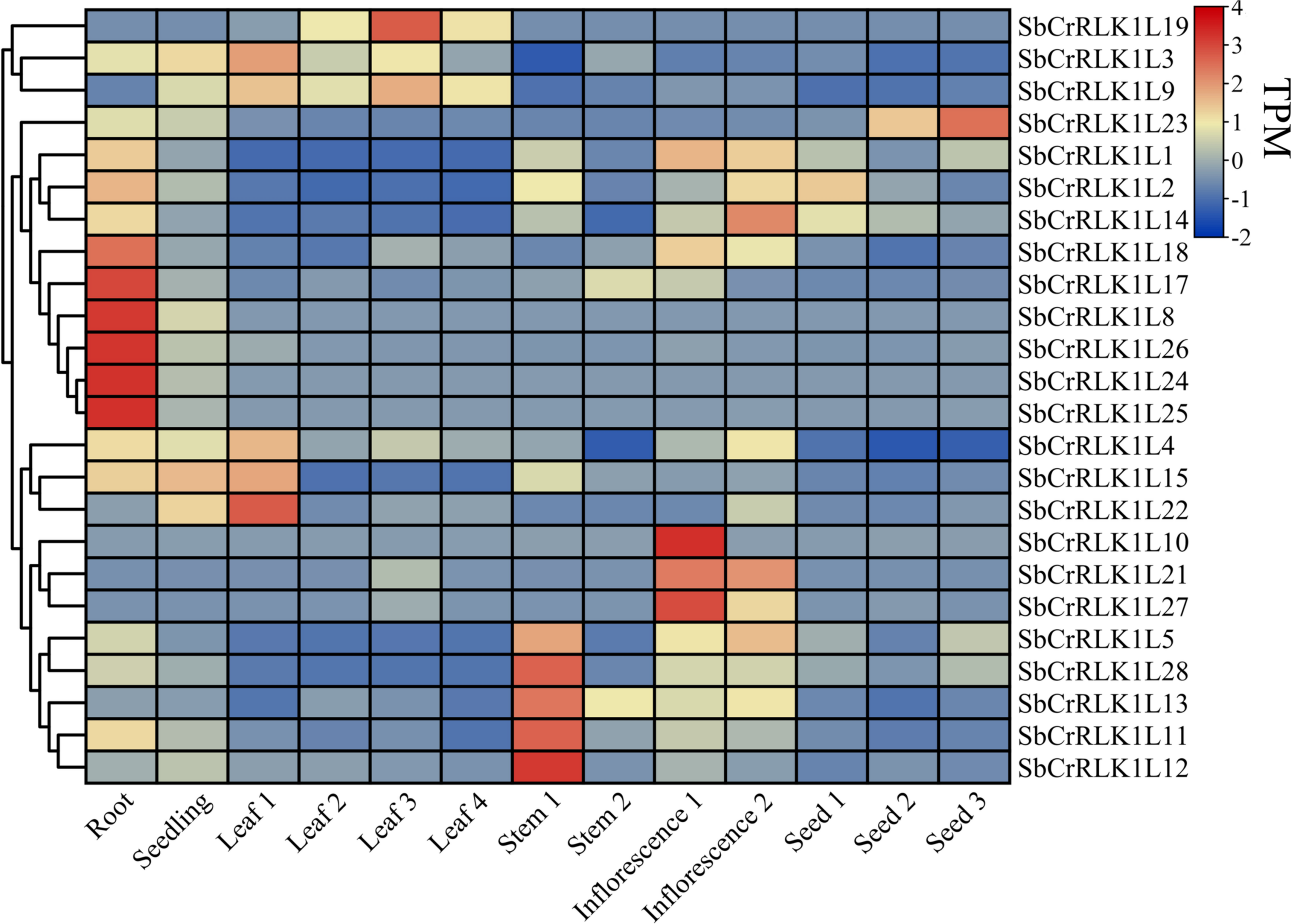
The heat map shows the expression level of the *SbCrRLK1L* gene in different tissues. Red and blue boxes indicate high and low expression levels of *SbCrRLK1L* genes.

To investigate the tissue-specific expression patterns of the *S. bicolor CrRLK1L* gene family, we systematically analyzed its transcript levels across multiple tissues—roots, stems, leaves, seedlings, panicles, and seeds—using publicly available RNA-seq data (**Figure 7** and **Supplementary Table 4**). The expression profiles revealed marked differences among tissues, with several genes exhibiting pronounced tissue specificity. For example, *SbCrRLK1L8*, *SbCrRLK1L17*, *SbCrRLK1L18*, *SbCrRLK1L24*, *SbCrRLK1L25*, and *SbCrRLK1L26* showed significantly higher expression in roots compared to stems, leaves, seedlings, inflorescence, and seeds. In contrast, *SbCrRLK1L10*, *SbCrRLK1L21*, and *SbCrRLK1L27* were predominantly expressed in inflorescences (**Figure 7**), while *SbCrRLK1L19* displayed the highest expression in leaves. These distinct expression patterns suggest functional diversification among *CrRLK1L* members in *S. bicolor* growth and development.

### Expression patterns of the *SbCrRLK1L* gene family under NaCl and PEG treatment

**Figure 8:**
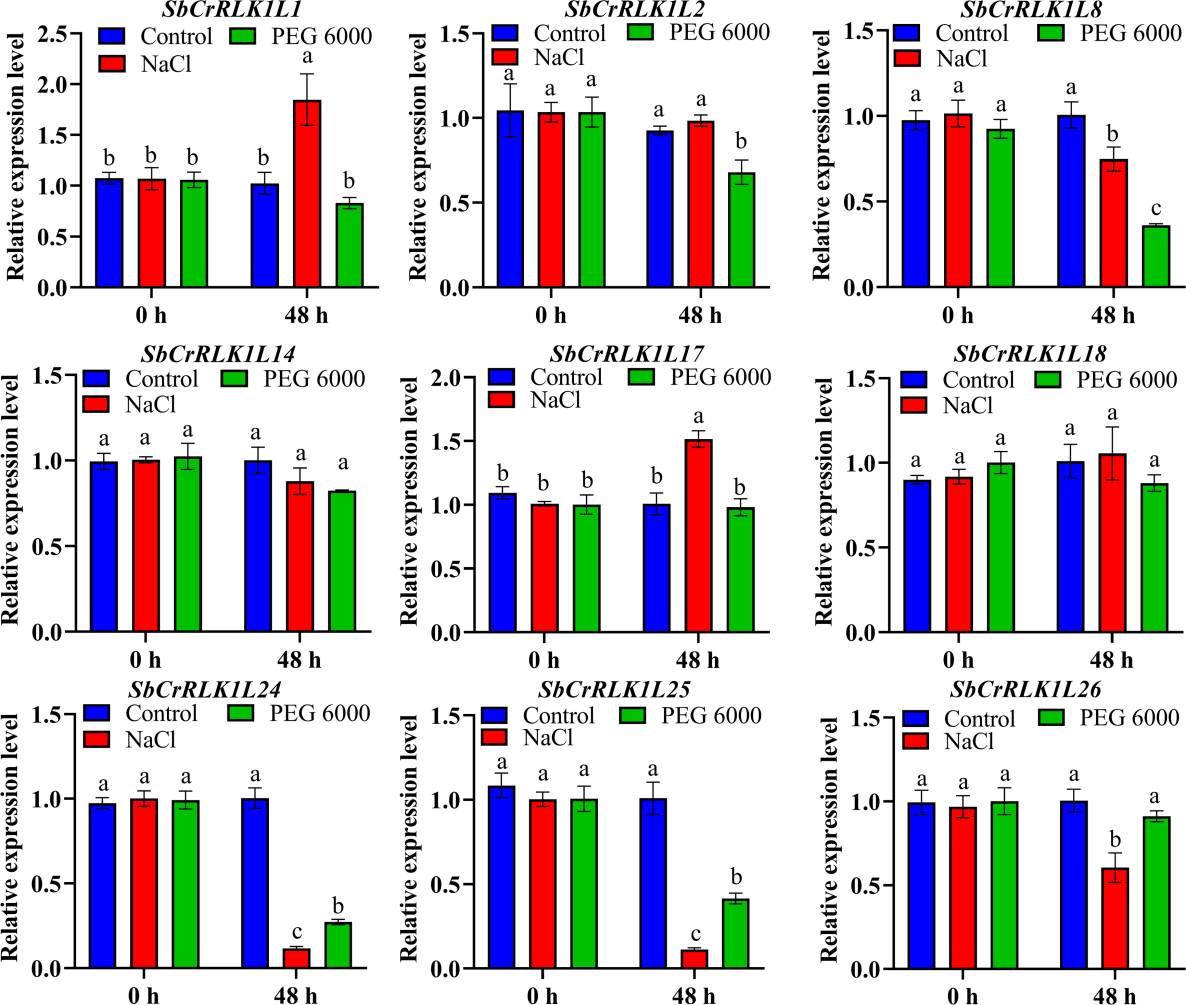
Expression patterns of *S. bicolor CrRLK1L* family genes under 20% PEG 6000 and 200 mmol/L NaCl stresses. The horizontal axis represents 20% PEG 6000 and 200 mmol/L NaCl treatments 0 h and 48 h, while the vertical axis indicates relative gene expression levels. Significant differences between treatments were determined by one-way analysis of variance (ANOVA), and error bars represent the standard error of the mean from three replicates.

To investigate the transcriptional responses of the *S. bicolor CrRLK1L* gene family to abiotic stress, we analyzed the expression patterns of selected members under drought and salt stress conditions using RT-qPCR (**Figure 8**). The results revealed distinct, stress-specific expression profiles among different genes. Under drought stress, the expression levels of *SbCrRLK1L8*, *SbCrRLK1L24*, and *SbCrRLK1L25* were significantly down-regulated. In contrast, salt stress treatment led to a marked up-regulation of SbCrRLK1L1and SbCrRLK1L17, while the expression of *SbCrRLK1L8*, *SbCrRLK1L24*, and *SbCrRLK1L25* remained consistently low, showing a similar suppression pattern to that observed under drought conditions. The divergent expression patterns suggest that members of the *S. bicolor CrRLK1L* family have undergone functional differentiation to mediate stress adaptation through specialized pathways. The specific up-regulation of *SbCrRLK1L1* and *SbCrRLK1L17* under salt stress implies their potential role in ionic homeostasis or osmotic adjustment. Conversely, the coordinated down-regulation of *SbCrRLK1L8*, *SbCrRLK1L24*, and *SbCrRLK1L25* under both drought and salt stress may represent a conserved strategy to modulate growth-related processes under resource-limiting conditions, potentially reallocating energy towards stress defense mechanisms.

## Discussion

### Comparative analysis of *CrRLK1L* gene families across different plant species

The *CrRLK1L* gene family encodes receptor-like kinases that play pivotal roles in plant growth, development, and stress responses through receptor-ligand signaling mechanisms (Shiu and Bleecker, 2001; Zhu et al., 2021; Franck and Westermann, 2018; Feng et al., 2018; Liu et al., 2024; Kanaoka and Torii, 2010; Stegmann et al., 2017; Solis and Quinto, 2021). While this family has been characterized in model plants such as *A. thaliana* and *O. sativa*, a comprehensive genome-wide analysis of *CrRLK1L* members in *S. bicolor* has been lacking. The present study performed a systematic bioinformatics-based identification of the *CrRLK1L* family in the *S. bicolor* genome. Our analysis revealed 28 *CrRLK1L* members, and further examination uncovered diverse physicochemical properties, conserved motifs, gene duplication events, and distinct expression patterns across tissues and under stress conditions. These findings provide a foundational resource for elucidating functional diversification and regulatory networks orchestrated by the *CrRLK1L* family in *S. bicolor*, with potential implications for improving crop resilience.

The number of *CrRLK1L* members varies considerably across plant species, reflecting differing evolutionary histories shaped by gene duplication events and selective pressures. Among dicot species, *A. thaliana* contains 17 members (Hématy et al., 2009), whereas diploid cotton species *G. raimondii* (D5) and *G. arboreum* (A2) harbor 44 and 40 members, respectively. Notably, allopolyploid upland cotton (*G. hirsutum* TM-1, AD1) possesses 79 members, illustrating WGD drives CrRLK1L gene family expansion (Niu et al, 2016). Other dicots—including *Nicotiana tabacum*, *Solanum melongena*, *S. lycopersicum*, and *Glycine max*—show intermediate numbers, ranging from 24 to 48 members (Li et al., 2022; Ma et al., 2023a, Ma et al., 2023b; Wang et al., 2021). In monocots, *O. sativa* contains 16 members, *Z. mays* (Wang et al., 2024) and *S. bicolor* possess 24 and 28, respectively, while hexaploid wheat exhibits a notably larger family size of 58 members (Gawande and Sankaranarayanan, 2024). This variation underscores the impact of polyploidization and lineage-specific duplication events on gene family evolution.

Notably, *S. bicolor CrRLK1L* family members are intermediate within the Poaceae family—larger than that of *O. sativa* but smaller than that of wheat—suggesting that *S. bicolor* has undergone specific genomic duplication events leading to moderate family expansion. This disparity likely correlates with wheat’s history of multiple rounds of allopolyploidy and localized segmental duplication events. Our analysis identified several segmentally and tandemly duplicated gene pairs within the *S. bicolor CrRLK1L* family, supporting the role of local duplication in shaping its genomic architecture.

Analysing the gene structure of the CrRLK1L family provides important clues for understanding its evolutionary relationships. We found that members of Group I and Group II high structural conservation in features such as the number members of Group III and Group IV typically have more exons and longer gene lengths, whereas the gene structures of Group I and Group II are relatively compact. Similar to the studies in *N. tabacum*, *S. melongena*, *S. lycopersicum* and *G. max* (Li et al., 2022; Ma et al., 2023a, Ma et al., 2023b; Wang et al., 2021). These structural differences may reflect the evolutionary differentiation undergone by each subfamily to adapt to different regulatory mechanisms and functional requirements. Notably, under drought stress treatments, the expression levels of SbCrRLK1L8/24/25 changed significantly. Compared with other family members, these three genes have a more complex structure, which may be related to their need for precise regulation in response to specific environmental signals.

### Evolutionary analysis reveals patterns in the evolution of the *CrRLK1L* gene family

The close phylogenetic relationship between *S. bicolor* and *O. sativa*, both members of the Poaceae family, is evident in the clustering of their *CrRLK1L* genes within the same major branches on the evolutionary tree, indicating a shared ancestral origin and potential functional conservation. In contrast, the *AtCrRLK1L* genes reside on distinct branches, reflecting the significant evolutionary divergence and functional differentiation that have occurred between monocot and dicot lineages. The internal structure of the *S. bicolor CrRLK1L* family, characterized by its subdivision into multiple subfamilies, suggests that genes within each subcluster may share not only a common evolutionary origin but also similar biological roles, a pattern consistent with observations in other plant species such as *Z. mays* and *soybean* (Wang et al., 2024; Ma et al., 2023b).

The expansion and diversification of the *S. bicolor CrRLK1L* family have been primarily driven by gene duplication events. Segmental duplication events have provided the genetic raw material for functional innovation, while tandem duplication has led to the formation of gene clusters on chromosomes, potentially enabling coordinated regulation and synergistic action in specific biological processes (Freeling, 2009; Magadum et al., 2013; Du et al., 2023). The evolutionary trajectory of these duplicated genes is shaped by selective pressures. The Ka/Ks ratios for *S. bicolor CrRLK1L* gene pairs are predominantly less than one, indicating that the family has undergone strong purifying selection (**Figure 6**). This selective constraint acts to preserve functionally important sequences by eliminating deleterious mutations, thereby maintaining the core functional integrity of the genes over long evolutionary periods.

### Correlation of expression patterns with plant growth and stress responses

The pronounced up-regulation of *SbCrRLK1L1* and *SbCrRLK1L17* under salt stress implies their involvement in maintaining ion homeostasis or modulating salt‐signaling pathways (**Figure 8**). This observation aligns with previous studies on *A. thaliana*, where the CrRLK1L member FERONIA (FER)interacts with RALF peptides and cell wall‐associated LRX proteins to regulate ion balance and cell integrity under salinity stress (Zhao et al., 2018). The conserved nature of this mechanism across species suggests that *SbCrRLK1L1* and *SbCrRLK1L17* may contribute *S. bicolor* salt tolerance through analogous pathways, potentially by influencing reactive oxygen species (ROS) metabolism or Na^+^/K^+^ equilibrium (Wang et al., 2014). On the other hand, the coordinated down-regulation of *SbCrRLK1L8/24*/*25* under both stresses may reflect a strategic resource reallocation strategy. These genes could function as repressors of growth‐related processes—such as cell elongation or photosynthesis—enabling the plant to conserve energy and redirect resources toward stress defense mechanisms. Notably, hormone signaling pathways likely serve as critical integrators in this regulatory network. The close association between early stress‐induced expression changes of *S. bicolor CrRLK1L* genes and phytohormone signaling pathways—particularly those involving ABA, JA, and auxin—suggests that *SbCrRLK1L* members may interface hormone cues with cell wall integrity monitoring systems, possibly through RALF peptide perception (Liu et al., 2023; Ge et al., 2019). This crosstalk could orchestrate a coordinated defense axis spanning from extracellular sensing to intracellular responses (Ge et al., 2019). Furthermore, genes that are suppressed under both stressors, such as *SbCrRLK1L8/24/25*, may normally support cell wall expansion and metabolic activity under non‐stress conditions.

Furthermore, our findings suggest that the promoter regions of the *SbCrRLK1L8*, *SbCrRLK1L24*, and *SbCrRLK1L25* genes contain elements that respond to drought. We also observed decreased expression levels of these genes under drought stress conditions. These results suggest that these genes play distinct yet crucial roles in the plant’s adaptation to drought stress. Our hypothesis is supported by prior studies in *O. sativa* (Jing et al., 2024) and *A. thaliana* (Murphy and De, 2014), which further substantiate the potential functional significance of these genes in tolerance mechanisms.

### Innovative contributions and limitations of the study

This study presents the first comprehensive bioinformatics analysis and expression profiling of the *S. bicolor CrRLK1L* gene family. By integrating multiple bioinformatics tools and databases, we systematically identified *S. bicolor CrRLK1L* genes, analyzed their physicochemical properties, determined their chromosomal locations, and constructed a phylogenetic tree. Furthermore, we examined gene structure, conserved motifs, gene duplication events, Ka/Ks ratios, promoter cis-acting elements, and expression patterns. Additionally, expression analysis under drought and salt stress offers important clues to the molecular basis of *S. bicolor* stress resistance, highlighting valuable genetic resources for stress-tolerant crop breeding. However, this study has some limitations. Currently, gene functions are primarily inferred from bioinformatics and expression analyses, lacking experimental validation. Future work could involve cloning genes of interest and conducting functional studies in transgenic systems to clarify the precise roles and mechanisms of *S. bicolor* CrRLK1L members. Interactions among family members and their crosstalk with other signaling pathways also remain to be explored, representing another critical direction for further research.

## Conclusion

In summary, 28 *SbCrRLK1L* genes were identified in *S. bicolor* genome. The phylogenetic tree revealed that *CrRLK1L* are divided into four Groups. Moreover, we found that the same Group of *SbCrRLK1L* contains similar gene structure and conserved motifs. Duplication event analysis indicated that the expansion of *SbCrRLK1L* gene family was primarily driven by multiple modes of gene duplication, coupled with strong purifying selection. The tissue expression pattern analysis showed *SbCrRLK1L8/17/18/24/25/26* may play an important role in root development. Furthermore, we found *SbCrRLK1L* may play a crucial role in the *S. bicolor* response to abiotic stresses, biotic stresses and nutritional deficiency defect. Under PEG6000 and NaCl treatment, *SbCrRLK1L1* showed an opposite expression pattern after, while *SbCrRLK1L8/24/25* showed a similar expression pattern. This study laid a foundation for further study on the function of *SbCrRLK1L1* genes in *S. bicolor*, and even other plants.

## Data Availability

Data are contained within the article or **Supplementary Material**. The datasets about the Public RNA-seq data during the current study are available in the SGMD (https://sorghum.genetics.ac.cn/SGMD/Expression.html).
